# Effect of Hydrofluoric Acid Etching Time on Titanium Topography, Chemistry, Wettability, and Cell Adhesion

**DOI:** 10.1371/journal.pone.0165296

**Published:** 2016-11-08

**Authors:** R. Zahran, J. I. Rosales Leal, M. A. Rodríguez Valverde, M. A. Cabrerizo Vílchez

**Affiliations:** 1 Prosthodontics Department, School of Dentistry, University of Granada, Granada, Spain; 2 Stomatognatic Physiopathology and Prosthodontics Research Group (CTS-974), University of Granada, Granada, Spain; 3 Instituto de Investigación Biosanitariaibs.GRANADA. University of Granada, Granada, Spain; 4 Applied Physics Department.School of Sciences.University of Granada, Granada, Spain; 5 Fluid and Biocolloid Physics Research Group, University of Granada, Granada, Spain; University of Akron, UNITED STATES

## Abstract

Titanium implant surface etching has proven an effective method to enhance cell attachment. Despite the frequent use of hydrofluoric (HF) acid, many questions remain unresolved, including the optimal etching time and its effect on surface and biological properties. The objective of this study was to investigate the effect of HF acid etching time on Ti topography, surface chemistry, wettability, and cell adhesion. These data are useful to design improved acid treatment and obtain an improved cell response. The surface topography, chemistry, dynamic wetting, and cell adhesiveness of polished Ti surfaces were evaluated after treatment with HF acid solution for 0, 2; 3, 5, 7, or 10 min, revealing a time-dependent effect of HF acid on their topography, chemistry, and wetting. Roughness and wetting increased with longer etching time except at 10 min, when roughness increased but wetness decreased. Skewness became negative after etching and kurtosis tended to 3 with longer etching time. Highest cell adhesion was achieved after 5–7 min of etching time. Wetting and cell adhesion were reduced on the highly rough surfaces obtained after 10-min etching time.

## Introduction

Ten-year implant survival rates are very high (>90%) [1]. However, some clinical situations (e.g., immediate loading, post-extraction implantation, sinus lift, systemic disease, poor bone quality, or osteoporosis) require improved implant surface characteristics to obtain a rapid cell response and early osseointegration and thereby prevent implant failure. The topography and wettability of the surface influence the first stages of bone formation, the quality of osseointegration, and the ability to retain the initial blood clot [2,3]. *In vitro* investigations at cellular and molecular level and histological studies have suggested that the surface properties of the implant affect bone formation at the interface by modulating the adherent cell phenotype [4].

Mechanical, chemical, or combined treatments have been developed to accelerate osseointegration. One of the most widely adopted approaches is to treat the implant with chlorhydric acid, sulfuric acid, or hydrofluoric (HF) acid with the aim of producing an irregular complex surface that enhances cell attachment and proliferation. HF acid treatment of a Ti implant surface has been found to increase cell adherence through bone-specific RNA expression [4] and to improve osteoblastic differentiation by increasing osteoblastic gene expression [5]. Osteocalcin and collagen type I gene expressions were also higher in HF acid-treated implants [6], and a positive correlation was reported between pull-out results and the amount of fluoride on the surface [7]. The incorporation of fluoride within titanium oxide film has been described [8] as a further advantage of HF acid treatment, although its presence was not found by some researchers [9,10,11].

Knowledge of the effects of etching kinetics on cell behavior is important to determine the optimal etching time to achieve maximum cell attachment. There has been limited published research on the impact of surface etching on the topography and osseointegration of titanium implants. Lamolle et al. immersed Ti implants in a weak HF acid solution (0.2% v/v) for up to 150 s and described time-dependent surface changes that were positively correlated with an improved biocompatibility; they detected an increase in roughness with longer immersion from 90 to 120 s and a more marked increase from 120 to 150 s. [12]. The authors did not study the effects of etching times on cell adhesion, and the optimal etching time for achieving maximum cell adhesion remains unknown. We have found no study on etching times longer than 150 s.

Biomaterial surfaces usually need to be hydrophilic in order to favor cell attachment, and the wettability of a solid surface can be quantified by measuring the contact angle. The most widely applied method is to quantify the contact angle of a single sessile drop on the target substrate. However, the contact angle of statically stable drops deposited with a handheld micropipette only yields information on low-energy domains of the surface, because the observed angle is closer to the advancing contact angle [13]. The receding contact angle is related to the high-energy domains of surfaces, such as metal oxides. The degree of hydrophobicity of a biomaterial surface should be determined from receding contact angles rather than from the contact angles of static drops. Hence, measurement of advancing and receding contact angles with dynamic methods based on the driven motion of menisci or drops provided more accurate data [14].

The objective of the present study was to investigate the effect of HF acid etching time on Ti topography, surface chemistry, dynamic wettability, and cell adhesion.

## Materials and Methods

### 2.1. Preparation of samples

Cylinders of commercially pure ASTM grade II Ti (Manfredi, S. Secondo di Pinerolo, Italy) was used. Each cylinder was cut into disks with a thickness of 2 mm, followed by a metallographic polishing protocol. The samples were polished using silicon carbide papers from grade 500, 800, 1200, 2000, to 4000 grit. They were then ultrapolished with a sequence of 1 to 0.3 to 0.05 μm alumina particles in a Beta grinder/polisher (Buehler, Munich, Germany). Next, samples were cleaned by immersion in an ultrasonic bath (Selecta, Barcelona, Spain), first in 50% ethanol (vol) (10 min) and then in distilled water (10 min).

The Ti disks were divided into 6 groups for different etching times: 0 min (no etching), 2 min, 3 min, 5 min, 7 min, and 10 min. Etching was performed by immersing samples in a solution of 10% (v/v) HF acid (Panreac, Barcelona, Spain) under magnetic agitation, with the Ti surface at right angles to the direction of the solution. Next, the etched samples were gently washed with distilled water and passivated with 30% (v/v) nitric acid (Panreac, Barcelona, Spain) for 3 min and were again ultrasonicated in 50% ethanol for 10 min and distilled water for 10 min, followed by drying at 37°C for 1 h. The sample was weighed before and after etching to calculate the percentage mass loss.

### 2.2. Surface topography

Topographies were acquired with a white light confocal microscope (PLμ Sensofar-Tech, Barcelona, Spain), examining three disks per group and acquiring three topographies per disk with an EPI x50 objective (scan size 292x214 μm^2^). The microscope software provided data on the topographic parameters [15]: arithmetic mean roughness (Sa), maximum relative height (Sp), maximum relative depth (Sv), Sp+Sv (St), root mean square roughness (Sq); skewness (Ssk), kurtosis (Sku), and the Wenzel factor (Sw). The fractal dimension (D_f_) was calculated from the topography data with the box-counting method [16], applying the fractal geometry equation N(d) = αd^-Df^, where N is the number of identical boxes needed to cover the entire surface [17], α is a geometric prefactor, d is the size of each box, and D_f_ is calculated from the slope of the log–log plot of the equation.

### 2.4. Microscopy

Three disks per group were examined three times under a Scanning Electron Microscope Leo 1430-VP (Carl Zeiss, Oberkochen, Germany). The morphology of each disk was evaluated from 3072×2304 pixel images acquired at a scan size of 205x155 μm^2^.

### 2.5. Surface chemical analysis

The surface elemental composition of another three disks from each group was determined by X-ray photoemission spectroscopy (XPS, Kratos Axis Ultra-DLD, Kratos Analytical, Manchester, UK) (scan size 300 μm x 700 μm).

### 2.6. Wettability

Wettability was evaluated in another three disks from each group. Before polishing, a central hole was drilled in each disk and a central Teflon cannula was inserted. After the polishing and treatment, the wettability was determined by measuring the dynamic contact angle using the ADSA-P technique (Axisymmetric Drop Shape Analysis-Profile). The advancing contact angle (θ_adv_) was determined from a sessile drop and the receding contact angle (θ_rec_) from a captive bubble, using Milli-Q purified water for the measurements. Measurements were taken in triplicate for each disk. The average of advancing and receding contact angles was obtained (θ_ave_) and used to calculate the Young contact angle (θ_Y_) as follows [18]

$${cos\theta }_{app}=S_{\text{w}}\;{cos\theta }_{\gamma }$$

where S_w_ is the ratio of the real to apparent surface area [19] and θ_app_ is the apparent contact angle.

### 2.7. Cell culture

Four disks from each group were immersed in osteoblast-like MG-63 cell medium for 24 h as reported elsewhere [20,21,22]. The experiment was performed in triplicate. Adhered cells per ml (n•100/ml) were then counted on three of the four disks with a cytometer (Ortho Diagnostic System, Raritan, Il, USA). The fourth disk was used to evaluate the cell morphology as follows: after the 24-h incubation period, the culture medium was removed and the disk was treated with 4% glutaraldehyde in PBS (pH 7.2) for 20 min to fix the cells, followed by dehydration in graded alcohols, immersion for 10 min in hexamethyldisilazane, air drying, and sputter-coating with gold palladium. Finally, the surface was examined under scanning electron microscope (Leo 1430-VP, Carl Zeiss, Oberkochen, Germany).

### 2.8. Statistical analysis

Data were analysed previously with a Kolomogorov-Smirnov test to confirm that were parametric data. Then, data were analysed with one-way ANOVA (surface treatment as main factor), followed by the post-hoc Student-Newman-Keuls multiple comparison test. p<0.05 was considered significant.

## Results

### 3.1. Surface topography

**Fig 1:**
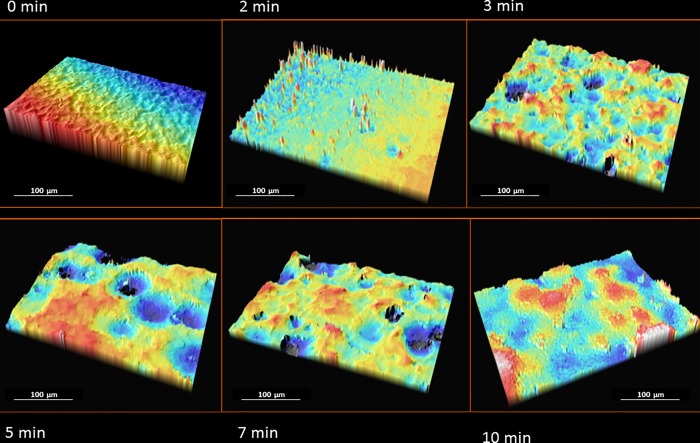
White light microscope micrographs (3D) of Ti surface at different immersion times (scan size 292x214 μm^2^).

**Fig 2:**
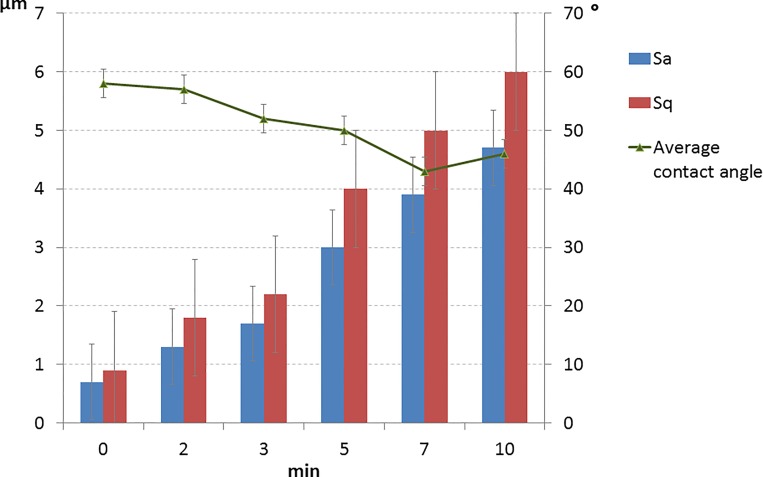
Graph showing the Sa, Sq y contact angle values after the different etching times.

Fig 1 depicts confocal images of Ti disks after different etching times (at the same scan size). Table 1 and Fig 2 exhibits results for the roughness parameters, showing that Sa and Sq roughness increased with longer etching time; values were similar between 5 and 7 min and highest after 10 min. Sp values increased up to 3 min of etching and then remained similar after longer times. Sv increased with longer etching time, being similar at 5 and 7 min and maximum at 10 min. St values were higher (vs. baseline) after 2 and 3 min of etching and highest after 5, 7, and 10 min, with no differences among them. The surface skewness (Ssk) was positive before etching and negative after etching in all etched groups. Surface kurtosis (Sku) decreased to a value of 3 with longer etching time, following a Gaussian distribution. The highest value was observed before etching, followed by 2 and 3 min (similar values) and then followed by 5 and 7 min (similar values). The lowest value was observed after 10 min immersion time. The Sw was increased after 2 and 3 min etching time, further increased after 5 min, and highest after 7 min. After 10 min of immersion, the Sw was reduced to the value found after 2 and 3 min. The dimensional fraction (D_f_) showed the same trend as observed for the Sw.

**Table 1:** Roughness, contact angle and cell adhesion data [mean (SD)].

Parameters		Etching time (min)					
		0	2	3	5	7	**10**
**Roughness parameters**	**Sa (**μ**m)**	0.7(0.1)a	1.3(0.2)b	1.7(0.7)b	3(1)c	3.9(0.9)c	4.7(0.8)d
	**Sq (**μ**m)**	0.9(0.1)a	1.8(0.7)b	2.2(0.4)b	4(1)c	5(1)d	6(1)d
	**Sp (**μ**m)**	3.1(0.1)a	8(3)b	11(2)c	11(2)c	13(2)c	14(2)c
	**Sv (**μ**m)**	-3.1(0.1)a	-13(4)b	-13(3)b	-17(4)c	-16(3)c	-20(3)d
	**St (**μ**m)**	6.2(0.2)a	21(4)b	24(3)b	28(5)c	30(3)c	34(5)d
	**Ssk (-)**	0.01(0.02)a	-0.5(0.5)b	-1(1)b	-0.6(0.3)b	-0.4(0.3)b	-0.5(0.1)b
	**Sku (-)**	18(5)a	10(4)b	9(3)b	6(3)c	5(1)c	3.2(0.4)d
	**Df (-)**	2(0.02)a	2.02(0.01)b	2.02(0.01)b	2.03(0.01)c	2.04(0.02)d	2.02 (0.01)b
	**Sw (-)**	1.00a	1.12(0.05)b	1.13(0.04)b	1.17(0.05)c	1.25(0.1)d	1.11(0.02)b
**Contact angle (degrees)**	**Advancing**	87(5)a	85(5)a	76(5)b	73(5)bc	63(4)d	70(4)c
	**Receding**	28(3)a	27(4)a	27(5)a	25(4)a	18(4)b	20(3)b
	**Average**	58(5)a	57(5)a	52(6)b	50(6)bc	43(6)d	46(4)dc
	**Young**	58(4)ab	61(3)b	56(4)bc	56(5)bc	54(5)cd	51(6)d
**Mass (**μ**g) & height (**μ**m) variations (%)**	**Mass lost**	0	0.021(0.004)a	0.042(0.005)b	0.061(0.006)c	0.093(0.004)d	0.123(0.005)e
	**Height loss**	0	0.007(0.002)a	0.012(0.003)b	0.015(0.005)b	0.065(0.002)c	0.085(0.006)d
**Cell adhesion (nx10**^**3**^**/ml)**		300(50)a	412(77)b	527(29)c	598(55)de	652(47)e	568(31)d
**Mass lost average:** 0.012% /min.							

SD: Standard deviation. Values with different letter in each row are statistically different (p<0.05).

### 3.2. Morphology

**Fig 3:**
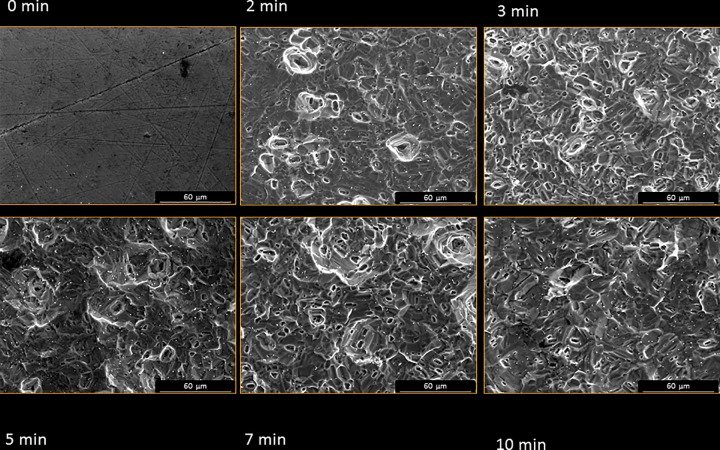
SEM micrographs of Ti surface at different immersion times (205x155 μm^2^ scan size).

Fig 3 depicts the SEM micrographs of the six groups. No etched Ti showed the smooth morphology observed in the non-etched sample. Acid etching produced angular stepped morphologies with micropores from 1.5 to 2.5 μm. More micropores can be observed after 3 min of etching time.

### 3.3. Surface chemical composition

XPS spectra (Table 2) showed that the surface chemistry of the non-etched surfaces differed from the early etched surfaces. Aluminum traces were found in non-etched samples from trapped alumina particles but were absent in the etched disks. Formation of fluoride on the titanium surface was only revealed at etching times of 2 min, while the native nitrogen content nearly decreased as the etching time. Both signals of Ti(2p) and O(1s) increased gradually from the non-etched group up to the 3 min-etched group. Adventitious carbon content was reduced as acid etching took longer. The surface composition remained stable after 3 min of etching time.

**Table 2:** Percentages of main chemical species found by XPS.

Etching time (min)	Ti (2p)	O (1s)	C (1s)	F (1s)	N (1s)	Al (2p)
0	7	27	60	0	3.5	2.5
2	4	29	62.5	0.5	4	0
3	15	53	30	0	2	0
5	16	52	30	0	2	0
7	15	45	39	0	1	0
10	17	47	35	0	1	0

### 3.4. Wettability

The advancing (θ_adv_), receding (θ_rec_), and average (θ_ave_) contact angles are reported in Table 1. θ_adv_, θ_rec_, and θ_ave_ decreased gradually as a function of etching time until 7 min and then rose at 10 min. The Young contact angle (θ_Y_) was reduced (vs. baseline) at 2 min and again at 3, 5, and 7 min. etching time (with similar values among them), with a further decrease at 10 min. θ_adv_ is related to roughness in Fig 2.

### 3.5. Cell culture

**Fig 4:**
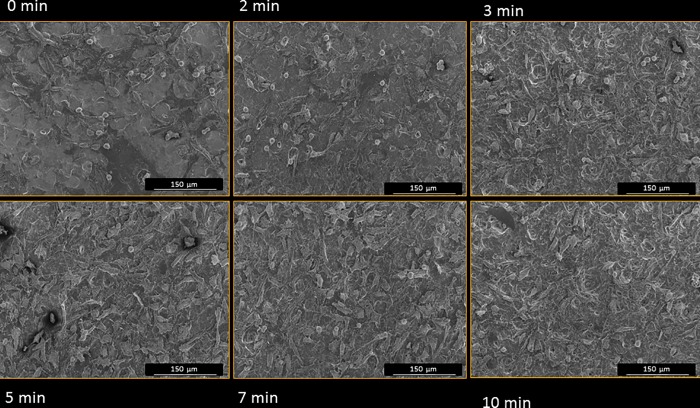
SEM micrographs of cells attached to Ti surfaces (205x155 μm^2^ scan size).

Cell adhesion data are compiled in Table 1. The cell adhesion rate increased as a function of etching time until 7 min and was decreased after 10 min. The maximum number of attached cells was detected after 5 and 7 minutes of etching time. Fig 4 depicts representative SEM micrographs of surfaces with attached cells after the different acid immersion times. Attached cell density was increased by acid etching and was highest at 3, 5, 7, and 10 min.

## Discussion

This study demonstrated that the duration of HF acid etching affects the topography, chemistry, wetting, and therefore cell adhesion of Ti surfaces. An etching time of between 5 and 7 min was found to achieve maximum cell attachment, which is crucial for rapid healing of the implant-bone interface. Etching-induced changes in the topography, wetting, and chemistry of the surface were responsible for this biological effect.

The surface roughness parameters measured (Sa, Sq, Sp, Sv, and St) showed a progressive increase with longer etching time up to 10 min (the maximum duration tested), consistent with the loss of Ti mass, which was proportional to the etching time. These topographic changes result from disorganization of the Ti crystalline structure produced by the acid etching. Although cell attachment appeared to be favored by increased roughness, it was reduced on the highly rough surface obtained after 10 min of etching time. It was previously observed that cell adhesion was lesser on surfaces that were etched and blasted than on those that were etched alone [22]. Likewise, a highly rough surface was found to reduce the bone-to-implant contact [23]. Osteoblasts have been found to adhere preferentially on nanometer and sub-micron structured titanium surfaces in comparison to smooth surfaces [24].

The skewness (Ssk value) was close to zero on the non-etched disks, i.e. the height distribution was symmetrical. A negative Ssk was observed on all etched groups, i.e., the surface contained holes or scratches rather than a predominance of peaks. No difference in Ssk value was found between etching times of 2 to and 7 min but it was higher, approaching zero, after etching for 10 min. It has been suggested that cells prefer a surface with negative Ssk but low roughness [12,22].

Sku values progressively reduced with longer etching time, from 18 on non-etched disks to 3.2 after 10 min of etching (Gaussian distribution). Sku values above 3 indicate sharp peaks (leptokurtic distribution), whereas values below 3 indicate more rounded peaks with wider shoulders (platykurtic distribution) [15]. The highest cell adhesion was found after 5–7 min, when Sku values were around 4–5. In a previous study, the highest bone-to-implant strength was observed in HF acid-treated Ti with a Sku value of 5.9, and cell attachment was increased at higher Sku values from 3.2 to 3.6; it was concluded that osteoblasts prefer smooth leptokurtic surfaces to Gaussian or platykurtic surfaces [12].

Etching produced a time-dependent increase in Sw values, which were highest after 5–7 min of immersion and were then reduced at 10 min. According to these findings, the real contact area was maximal after 5–7 min of HF acid etching, with a reduction in this area after a longer etching time. Cell attachment is known to be higher with larger contact area [25]. Etching for 10 min reduced the area ratio and cell attachment.

Df values increased progressively with longer etching time except at 10 min, when they were reduced. The fractal dimension is considered the best index of surface disorder [26] because the results are independent of the observation scale [17], although a complete description of a surface also requires data on the kurtosis and skewness and information from TEM images. Fractal surfaces with the same fractal dimensions but distinct lacunarities can have widely different characteristics. We observed a positive relationship between Df and cell attachment values, as previously reported [22].

XPS findings revealed a progressive increase in the surface contents of Ti and O and a decrease in C content with longer acid etching up to 3 min, after which the chemical surface composition remained stable. These results are in agreement with previous reports [7,11,12,27]. A reduction in C, which likely derives from atmospheric CO_2_, has been found to increase the energy compatibility of Ti surfaces [11]. This contributes to explaining the higher cell attachment found after etching times beyond 3 min. Surface O is increased because the HF acid etching removes contaminated layers, enhancing surface reactivity and forming a thick layer of TiO_2_. This layer, which is thicker at the beginning of acid etching, has been reported to play a key role in protection [11] and to increase cell adhesion [28]. F was only observed after 2 min of HF acid immersion, when the surface TiO_2_ first reacts with fluoride ions from the solution. This layer is removed at longer etching times due to the strong corrosive effect of HF acid on Ti oxide [12]. It has been estimated that the passive TiO2 layer is dissolved after etching with 0.2% HF acid for approximately 150 s. [12], after which time F cannot be detected, as also found by other researchers [9,11,12]. The effects of surface F on cell adherence have yet to be established [4,5]. All studies of F-containing surfaces have prepared these surfaces by etching a smooth Ti surface with HF acid, which also produces a rougher surface [6,7,8,11,12]. There is a need to compare between the presence and absence of F on surfaces of a similar roughness in order to elucidate the effect of F on cell adherence.

Wetting was reduced with longer etching times until it reached its lowest value at 7 min, followed by an increase after 10 min. The wettability of a surface is considered as a relationship among three phases (liquid, solid and gas) and characterized by applying the Young-Laplace equation [29]. In the present study, contact angle variations were due to modifications in the solid phase, given that the liquid and gas phases were constant. The solid can be chemically or topographically (irregularities or roughness) modified. For the same titanium surface chemistry, surface energy and wetting are drastically altered on nanometer (less than 100 nm)) and sub-micron (later than 100 nm) *versus* smooth surface structures [30]. Irregularities of the solid surface influence contact angle measurements. We used a dynamic contact angle technique in which the advancing and receding contact angles were obtained. This approach is essential for chemically heterogeneous and rough Ti surfaces with contact angle hysteresis [30,31].

Roughness and wetting are related in the Wenzel equation [24], which gives the ratio of actual to apparent or projected area, and θ_app_ refers to the contact angle on the real roughened surface, while θ_Y_ (Young contact angle) refers to the ideal smooth surface. Accordingly, if the contact angle measured on a smooth surface is less than 90°, it is further decreased by roughness, while if it is greater than 90°, it is increased. In this study, the contact angle measured on the non-etched polished Ti surface or smooth surface (θ_app_) was less than 90° and was reduced (θ_Y_) by etching times up to 7 min but was increased after 10 min of etching, despite the greater roughness. Initial contact angle variations were also due to chemical modifications. The Young contact angle was increased due to the chemical changes that resulted from acid etching for 2–3 min (removal of Al, reduction in C, and brief generation of F) and then decreased after 3 min (stable signals of Ti, O and C). The contact angle remained relatively stable after 3, 5, and 7 min of etching, indicating that the variations in wetting were attributable to topographic factors. However, there was a reduction in the angle after 10 min of immersion, indicting a change in surface energy. In contrast to the prediction of the Wenzel equation, not only the greatest roughness but also the least wetting was observed after 10 min of etching. We can conclude that the most influential roughness parameters are related to the form and distribution of peaks [32]. When peaks are higher (increased Sp, Sv and St values), an increased contact angle can therefore be observed on a very rough surface. It has been found that the degree of cell spreading markedly increases with a certain rise in roughness but is then diminished by further increases in roughness [31]. Hence, no greater improvement in wetting is expected for highly rough surfaces, as confirmed by the reduction in wetting observed after 10 min of HF acid etching in the present study.

Other studies reported a contact angle reduction (wetting increase) after acid etching [22,33], but there has been little investigation of the contact angle as a function of Ti etching time. One investigation found that the wetting was increased and the contact angle reduced with longer etching times, but they used static contact angle measurements [12].

The wettability of an implant surface plays a key role in its success through its effect on protein adsorption and therefore on cell attachment and tissue integration at the bone-implant interface [24,33,34]. Initial reactions of the biomaterial with water and proteins are influenced by surface wetting [32,34]. Cell adhesion and wetting were related in this study, as previously reported [22,33,35].

## Conclusion

From the data obtained in this study, the conclusions can be summarized as follows:

1. HF acid treatment of Ti surfaces modifies their chemical composition, which becomes stable after 3 min. of etching.
2. Skewness becomes negative after etching, and kurtosis tends to 3 with longer etching time. Roughness and wetting values increase with longer etching time up to 7 min; however, after 10 min of etching, the wetting is decreased despite the increased roughness.
3. Surface modifications influence cell attachment. Highest cell adhesion is achieved after 5–7 min of etching time. Cell adhesion is reduced on the highly rough surfaces obtained after 10 min etching time. According to these findings, the optimal duration of HF acid treatment of Ti implant surfaces is 5–7 min.

## Supporting Information

S1 Table Relevant contact angle data. (TXT)

S2 Table Relevant roughness contact angle data. (XLSX)
